# Experimental Demonstration of Adaptive Infrared Multispectral Imaging using Plasmonic Filter Array

**DOI:** 10.1038/srep34876

**Published:** 2016-10-10

**Authors:** Woo-Yong Jang, Zahyun Ku, Jiyeon Jeon, Jun Oh Kim, Sang Jun Lee, James Park, Michael J. Noyola, Augustine Urbas

**Affiliations:** 1 Air Force Research Laboratory, Sensors Directorate, Wright-Patterson AFB, 45433, USA; 2 Sensor APEX, University of Dayton Research Institute, Dayton, 45469, USA; 3 Air Force Research Laboratory, Materials Directorate, Wright-Patterson AFB, 45433, USA; 4 Korea Research Institute of Standards and Science, Division of Convergence Technology, Daejeon, 305-340, Korea

## Abstract

In our previous theoretical study, we performed target detection using a plasmonic sensor array incorporating the data-processing technique termed “algorithmic spectrometry”. We achieved the reconstruction of a target spectrum by extracting intensity at multiple wavelengths with high resolution from the image data obtained from the plasmonic array. The ultimate goal is to develop a full-scale focal plane array with a plasmonic opto-coupler in order to move towards the next generation of versatile infrared cameras. To this end, and as an intermediate step, this paper reports the experimental demonstration of adaptive multispectral imagery using fabricated plasmonic spectral filter arrays and proposed target detection scenarios. Each plasmonic filter was designed using periodic circular holes perforated through a gold layer, and an enhanced target detection strategy was proposed to refine the original spectrometry concept for spatial and spectral computation of the data measured from the plasmonic array. Both the spectrum of blackbody radiation and a metal ring object at multiple wavelengths were successfully reconstructed using the weighted superposition of plasmonic output images as specified in the proposed detection strategy. In addition, plasmonic filter arrays were theoretically tested on a target at extremely high temperature as a challenging scenario for the detection scheme.

Infrared (IR) spectral imagers^1^,^2^,^3^ have been widely employed to detect the spectral content of radiation from various objects at several wavelengths of interest. A conventional spectral imager collects a series of images with dispersive elements such as a filter wheel in front of an imager viewing a scene. The captured images contain narrowband spectral responses of objects at specific wavelength locations. By employing a data processing technique, spectral images are aligned in a three-dimensional array of values (termed a “data cube”) in order to construct a spectrally-resolved image of the object, which is then analyzed to identify, for example, the materials compositions of the object in a scene, and enable discriminating among different classes of materials.

A new class of IR sensing techniques, termed algorithmic spectrometry^4^,^5^,^6^,^7^,^8^, has been developed as an alternative to traditional approaches. This novel approach is particularly designed for pixelated IR sensors having spectrally coarse and overlapping spectral responsivities of the pixel types. The idea of the algorithmic spectrometer is to reconstruct the spectrum of any unidentified object of interest without utilizing any intervening spectral filters or equivalent optics in the optical train. A key step is to find the optimal set of weights by the projection algorithm^9^, which enables the synthesis of a desired spectral shape (with a specified center wavelength and bandwidth) onto the linear space spanned by the sensor’s spectral responsivities. As a result, the outcome from the projection step is a synthetic spectral responsivity, composed of a mathematically defined artificial, arbitrary and hypothetical bandpass filter which best approximates a desired spectral filter shape. This synthetic spectral responsivity then serves as a means for reconstructing the radiation of an unidentified object at a wavelength of interest (i.e., spectral signature). Sensing in the transformed space (spanned by the synthetic spectral responsivity) is highly flexible since synthetic spectral responsivity can be arbitrarily changed in shape, center wavelength, or bandwidth without utilizing any hardware or optical elements. In addition, the synthesis of the IR sensor’s responsivity naturally leads to significant data reduction since the number of different sensor bands defined by the pixel responses are significantly fewer in number than in traditional spectral sensing bands.

In the human visual system^10^,^11^, a similar mechanism can be observed in terms of recognizing the spectral contents of an object, resulting from the interpretation of the spectral response of three different types of color-sensing elements. Analogous to the functionality of the lens and the film in a camera, an image of the visual world is created on the retina through the cornea. The retina is the inner coat of the eye consists of several layers of neurons. Among them, the photoreceptor cells are light sensitive elements, mainly of two types: rods and cones. Particularly, cones support daytime vision and the perception of color with three classes, often referred to as the long L peak in the red, the medium M peak in the green, and the short S peak in the blue. These three L, M and S cones display different, but partially overlapping response curves. This corresponds with our IR sensor’s different pixel outputs or spectral responsivities. Despite the overlap in individual cone spectral responsivities that results in redundant wavelength sensing, the human visual system can respond to variation in color in different ways due to trichromatic vision. The brain would not be able to distinguish different colors, if the input would be from only one type of cone cell, simply due to lack of information. With at least two or three types of cones, the brain, learning through experience, can compare the signals from each type of cone cell, and thereby determine color, i.e. both the intensity and wavelength of the light, using combinations of neighboring cell responses.

In recent years, the physical phenomenon of surface plasmon (SP) resonance^12^,^13^,^14^,^15^ has been demonstrated to have a high potential for use as multispectral (MS) sensing elements or couplers for IR imagers. This is due to the ease of tuning the resonance wavelength, and providing the ability to design transmission and reflection properties, thereby offering different spectral shapes for a sensor’s responsivities. The change in SP resonance wavelength is mainly correlated to the structural periodicity. In our recent publications^14^,^15^,^16^,^17^,^18^, a 2-D array of gold (Au) circular holes (CHA) was used as our SP resonance structure and the periodicity of CHA was varied to tune the SP resonance wavelength. Corresponding spectral curves through various CHAs have shown spectral shifts in the SP resonance, as well as partial overlaps between neighboring curves similar to the response spectra of cone cells in the human eye.

The IR retina concept (algorithmic spectrometry)^19^,^20^,^21^ was then implemented in order to decorrelate the overlapping spectra of individual CHAs through weighted combinations of the sensed outputs (similar to principal component transformation) just as the human eye does. Results drawn from the algorithm successfully reconstruct the spectral signatures of an object at desired wavelengths, along with a reconstructed spectral bandwidth beyond the inherent characteristics of original CHAs. Additionally, the IR retina concept was implemented as a SP-based superpixel^18^, reconstructing the object’s signature both spatially and spectrally at a location in the scene. Hence, forming different SP arrays over a single imaging plane in conjunction with data-processing algorithm enables real time, spatially resolved collection of the multispectral information content of imaged scenes.

For this paper, we present the first experimental demonstration of this reconstruction using an array of SP spectral filters to measure the radiant power from an unknown source in a scene in the long wave IR (LWIR) region. The LWIR portion of the electromagnetic spectrum is the region of interest for our specific effort, since objects near room temperature emit thermal radiation in this range, allowing imagery that is largely independent of illumination conditions. Furthermore, to clarify the difference between our study and previous works reported in refs 18, 19, 20 it is worth noting that the proposed plasmonic sensing strategy has not been fully realized prior to this work, i.e., the implantation and performance analysis of IR retina concept was mostly carried out either with a single pixel IR device (experiment: spectrally-reconstructed target’s signature)^19^,^20^ or with a SP-based superpixel (simulation: spatially- and spectrally-reconstructed target signatures)^18^.

The paper consists of three main sections. The first section discusses the design and fabrication of the SP spectral filter arrays based upon a CHA structure with resonances in LWIR range (8–12 μm). The second section is about the post-processing strategy for LWIR object detection based on the algorithmic spectrometry concept. The last section is a performance analysis of the proposed strategy, using examples of reconstructing the irradiance of an IR source (blackbody) as well as a metal ring object. Furthermore, a simulation study is carried out for potential application of SP spectral filter arrays to detect objects at extremely high temperature in a scene that would typically saturate IR detector systems.

## Results

### Design of an array of SP spectral filters

Prior to the design of the surface plasmon (SP) resonance structures, three requirements were specified by the algorithmic spectrometer for the object detection in long-wave IR (LWIR). These were SP resonance wavelengths, the number of SP resonances within the wavelength range of interest, and the bandwidths of the sensor’s responsivities via SP resonance structures. A brief description of the algorithm will be provided in the following section. The SP resonance wavelengths were set between 7.5 and 11 μm, the number of resonances was set to be at least 4 or greater, and the the full-width at half maximum (FWHM) bandwidth required was set to 2 μm or less. Using these requirements as the design criteria, the SP structural simulation was carried out for a single Au layer perforated with a 2-D square array of circular holes (CHAs). Finite integration technique^22^ and rigorous coupled wave analysis^23^ based simulations were used, and results obtained from both techniques were comparable.

**Figure 1:**
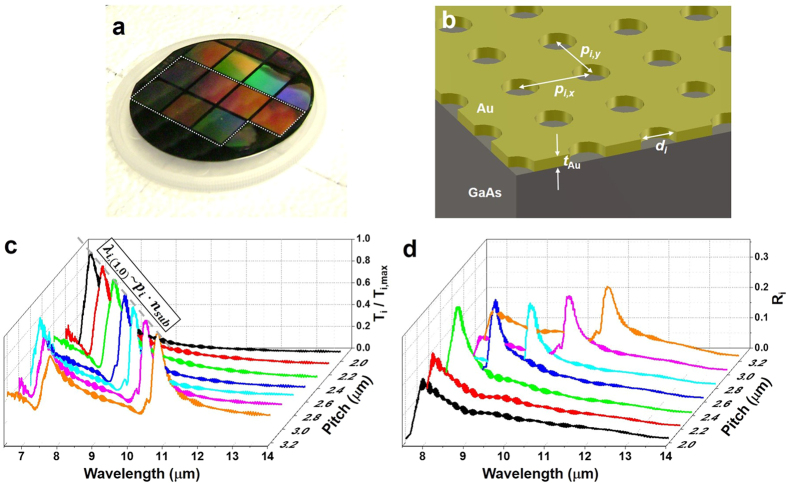
A photograph of the fabricated array of SP based spectral filters, illustration, transmission spectra and LWIR spectral responsivities via SP filter arrays. (**a**) Photograph of SP spectral filter arrays which consists of seven different CHA structures (Note: The region enclosed by white dotted line shows the SP filters utilized for the experiment). (**b**) Geometry and dimension of CHA structure for each SP filter, 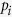 = 2.0 μm ~ 3.2 μm with 0.2 μm step;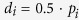; 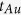 = 0.1 μm. (**c**) FTIR-measured transmission spectra (T_*i*_) through SP filter arrays shown in **a**. The transmission measurement was carried out at normal incidence to the sample then normalized to the maximum value. (**d**) Corresponding LWIR spectral responsivities of collected outputs at the camera via SP filter arrays.

For all SP structures, the orthogonal pitches of CHAs, *p*_*i,x*_ (pitch along *x*-direction) and *p*_*i,y*_ (pitch along *y*-direction) are both fixed at *p*_*i*_ (*p*_*i*_ = *p*_*i,x*_ = *p*_*i,y*_), where *p*_*i*_ is varied from 2.0 μm to 3.2 μm with a step of 0.2 μm and the subscript *i* indicates the periodicity as shown in Fig. 1. The SP resonance transmission peak-wavelengths are expected to change with the pitch *p*_*i*_; the first-order 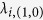 and second-order 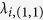 SP resonances attribute to the nearest- and next nearest-neighbor-hole coupling, respectively. Their resonant transmission peak-wavelengths for normal incidence can be calculated using the momentum matching condition^12^ 
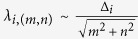
, where 
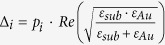
 and *m*, *n* are integers representing the SP coupling order. The ratio *d*_*i*_/*p*_*i*_ and Au thickness *t*_*Au*_ are fixed at 0.5 and 100 nm, respectively. As a result, the first-order SP resonance transmission was peaked at ~6.9 μm (*p*_*i*_ = 2.0 μm) and gradually red-shifted as *p*_*i*_ increased. Note that the FWHM for each CHA’s transmission spectrum was found to be around 0.6 μm.

Based on simulation results, we fabricated the array of SP based spectral filters on double-side, polished, 2-inch, semi-insulating GaAs. A brief description of the fabrication steps is provided as follows: (1) Standard photolithography was employed to produce periodic circular post array in the photoresist layer; (2) 5-nm-thick adhesion layer of titanium (Ti) and 100-nm-thick layer of Au were deposited using e-beam evaporation, followed by a liftoff processing (CHA structure as displayed in Fig. 1b); (3) Finally, a 200-nm thick Ti layer was deposited to isolate the array of SP spectral filters from incident IR radiation (to block the unwanted area from incident IR light), which leads to the final structure of SP spectral filter arrays as shown in Fig. 1a. A further description is included in the Methods section.

The transmittance of the fabricated SP spectral filters (Fig. 1c) was measured by using an FTIR spectrometer under an atmosphere of nitrogen purged at 30 ~ 40 sccm, which prevents IR absorption by components of ambient air. See Methods for more details. The measured transmission spectrum (T_*i*_) determines how much IR beam is incident on the LWIR camera^24^ through each SP filter. As a result, the spectral responsivity (*R*_*i*_), i.e. the camera output due to IR beam transmitted via each SP filter is established by relating T_*i*_ to the relative responsivity of a LWIR camera *R*_*LWIR*_ as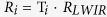. All spectral responsivities (Fig. 1d) transmitted via an array of SP filters were obtained after repeating the FTIR measurements. Note that in Fig. 1c, it can be clearly seen that the measured 1^st^ order SP resonance peak redshifts from 6.9 μm to 10.8 μm as the pitch is increased from 2.0 μm to 3.2 μm, as previously expected. More details of Fig. 1c,d are included in the Supporting Information section.

### Target detection strategy using SP filtered outputs

**Figure 2:**
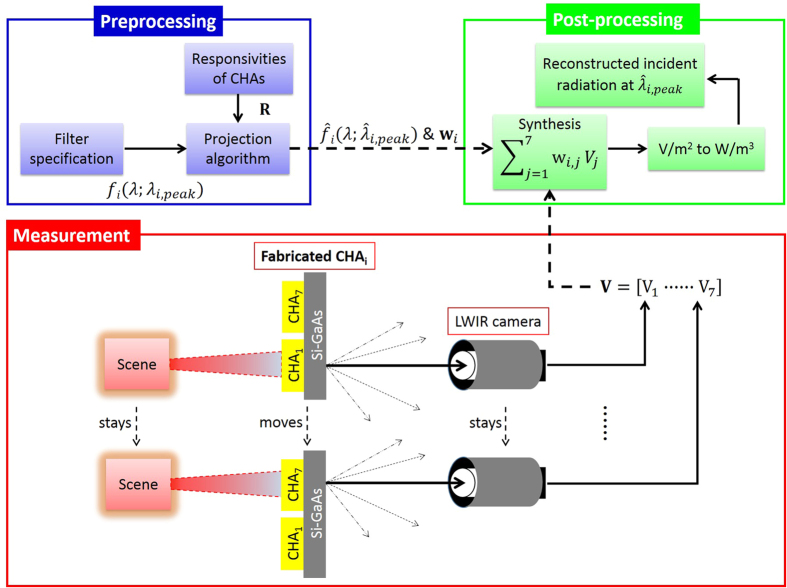
Block diagram illustrating a target detection strategy using an array of seven SP filters to identify the incident power from an unknown source at a specific wavelength. The *i*^th^ SP filter in the array is labeled as CHA_*i*_. The strategy comprises the preprocessing, measurement and post-processing.

Our strategy is based upon the principle that by performing a weighted linear combination of pixel level signal responses through the SP filters, the incident power per unit area from an unknown object at a specific wavelength can be identified. As illustrated in Fig. 2, the identification process is carried out in three major steps: (1) Pre-processing; (2) Measurement, and (3) Post-processing. A detailed description follows. The pre-processing involves the specification of desired spectral filters (i.e., shape, center wavelength and bandwidth) and the calculation of projection weights. It is worth pointing out that the pre-processing step is performed independently of source information. Once filter parameters are set, the projection algorithm estimates a filter shape in a minimum mean square error sense by calculating a set of weights in which each weight corresponds to individual spectral responsivity associated with CHA_*i*_. By drawing freely from ref. 19, a set of weights **w**_*i*_ corresponding to the desired spectral filter ***f***_*i*_ (a matrix form of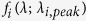) is computed according to the following equation,


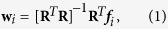


where **R** is the vector of spectral responsivities *R*_*i*_ for incident power transmitted via SP filters. A description of the projection algorithm for **w**_*i*_ is available in the Supporting Information section.

A linear combination of responsivity spectra in **R** with **w**_*i*_ optimally approximates the desired filter *f*_*i*_ with a synthetic spectral filter, 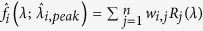 where *w*_*i,j*_ and *R*_*j*_(*λ*) are the *j*^th^ component in **w**_*i*_ and **R**, respectively. This filter 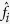 is what we would employ for extracting the spectral content of a source from the scene at a specified wavelength (
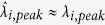
). The calculation is repeated to obtain the weight vector **w** (i.e. a collection of **w**_*i*_) for the remaining spectral filters of interest in the filter set **F**, yielding a group of weight sets. In the measurement, the output (*V*_*i*_) is taken when each SP filter (i.e., CHA_*i*_) is exposed to a test scene with a radiating source of interest. In the post-processing step, a collection of sensed outputs,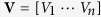, where *n* is the number of SP filters applied is linearly synthesized with weights **w**_*i*_. This synthetic output is then transformed into the power per unit area per wavelength (W/m^3^) according to the relationship
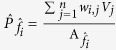
, where 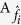 is a relating (normalizing) factor and is equal to the area under measured responsivity curve via synthetic filter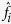. This reconstructed power 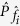 corresponds to the property that we would have probed using a broadband LWIR camera looking at the same source directly through the synthetic filter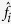. To reconstruct the incident radiation at multiple peak-wavelengths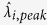, the post-processing needs to be repeated using different weight sets **w**_*i*_. By doing so, multiple sets of weights can be determined corresponding many wavelengths that can be independently reconstructed from a single measurement set.

### Test scenario I: Multispectral reconstruction of blackbody radiation

As a proof of concept for our detection strategy explained in previous section, we specified a test scenario as follows. The blackbody was used as an unknown test source and set to radiate at 300 °C. For measurement, we placed the fabricated array of seven SP spectral filters, 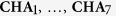
(as shown in Fig. 1a) in front of the LWIR camera looking at a blackbody source. Experimental outputs 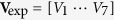 were then collected at the camera as each SP spectral filter, 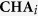 was exposed to the scene (moving from 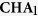 to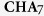), as illustrated in Fig. 2.

**Figure 3:**
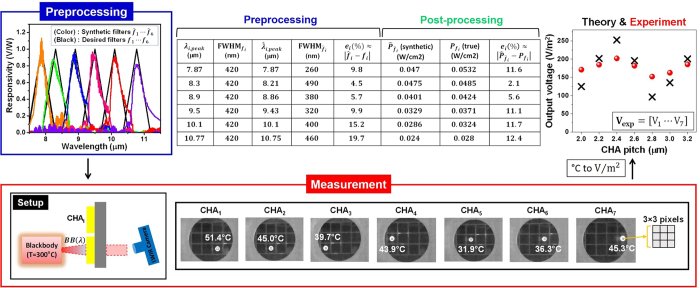
Multispectral reconstruction of blackbody (used as an unknown, simple thermal source) information using the detection strategy described in previous section. Preprocessing: The desired triangular shaped filters (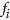 where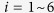: in black) with a fixed FWHM of 420 nm and synthetic filters (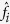 where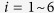: in color) with FWHMs varying from 260 nm to 490 nm (differences in peak-wavelengths and FWHMs are 
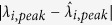
 = 0, 90, 40, 70, 0, 20 nm and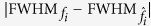 = 160, 70, 60, 100, 20, 40 nm, respectively). The projection errors 
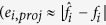
) are calculated with respect to the desired triangular shaped filters, ranging from 4.5% to 19.7%. Measurement: Seven sensed outputs in °C (is converted into V/m^2^) as a blackbody radiates through each CHA_*i*_. Measurement was repeatedly performed as moving from one CHA_*i*_ to another sequentially. Post-processing: Reconstructed blackbody radiation 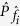 at six different wavelengths 
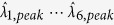
 and the reconstruction errors 
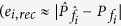
) as compared to the reference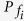. Corresponding weight sets 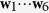 are used to reconstruct the multispectral property of an unknown thermal source (blackbody) in the LWIR. The table summarizes all the preprocessed and post-processed results by our detection strategy in order to characterize the unknown source.

In the top right panel of Fig. 3, measured outputs **V**_exp_ in V/m^2^ through SP filter arrays are plotted as a function of CHA’s pitch for each SP filter since the pitch variation is the main factor to produce seven SP-filters (corresponding to designed SP-spectral responsivities as previously explained). In Fig. 3, the measured outputs **V**_exp_ are also plotted with theory **V**_theo_ for validation (Calculation of **V**_theo_ is included in Supporting Information section). As observed in Fig. 3, the overall trend as a function of CHA’s pitch is consistent between theory and experiment. However a discrepancy between **V**_exp_ and **V**_theo_ is possibly due to two reasons: one is the imperfections in the fabrication of SP filter arrays and the other is **V**_theo_ was simulated under normal incidence while **V**_exp_ was measured with the LWIR camera which was slightly angled to face the SP-filter arrays to avoid the significant reflection at the surface.

While the blackbody source information is assumed unknown, the objective is to spectrally reconstruct the incident radiation of the source at multiple wavelengths in order to establish what it is. For this scenario, we specified six desired triangular filters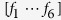 in the preprocessing step of our strategy for multispectral reconstruction of an unknown source. Six desired filters have peak-wavelengths at *λ*_*i,peak*_ = 7.87, 8.3, 8.9, 9.5, 10.1, 10.77 μm, respectively and each has a FWHM of 420 nm as shown by solid black lines in the top left panel of Fig. 3. To realize these desired filters *f*_*i*_, six synthetic filters 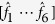 were designed by finding corresponding weight vector **w** (a set of **w**_*i*_) according to the solution in equation (1) for the projection algorithm. By comparison between *f*_*i*_ and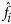, the errors in peak-wavelengths 
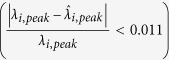
 and FWHMs 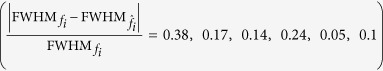 were reasonably low. In addition, the overall errors described by the shape matching error *e*_*i*_ (i.e., 
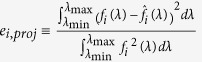
reported in ref. 18) in the table of Fig. 3 were also acceptable having a minimum of 4.5% for 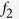
and a maximum of 19.7% for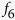.

In the post-processing step, the measured SP outputs in **V**_exp_ were multiplied by pre-determined weights in each **w**_*i*_ then summed (i.e.,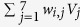), yielding a set of synthetic outputs. Each synthetic output was transformed into the value in W/m^3^ using a relating factor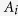, hence reconstructing the radiation of a blackbody source at a wavelength of 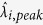 (i.e., 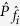). The post-processing step was repeated for entire set of weights 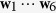 to reconstruct the source radiations at six different peak wavelengths 
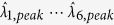
. The table in Fig. 3 lists the reconstructed blackbody radiations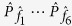 after post-processing as well as comparison to the ground truth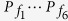. Each reference 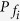 corresponds to the reconstructed radiation using an ideal triangular filter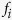. More detail about the ground truth are available in the Supporting Information section.

We achieved a reconstruction error (i.e.,
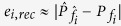
) as low as around 2% at 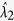 = 8.21 μm. It is important to mention that the reconstruction errors greater than 0 are attributed to a couple of factors: first, is the imperfection in the projection of a desired spectral filter 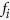 since the projection error 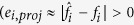) and, second, is the experimental uncertainty in **V**_exp_ as compared to V_theo_.

### Test scenario II: Reconstructed multispectral LWIR images of a metal ring object

**Figure 4:**
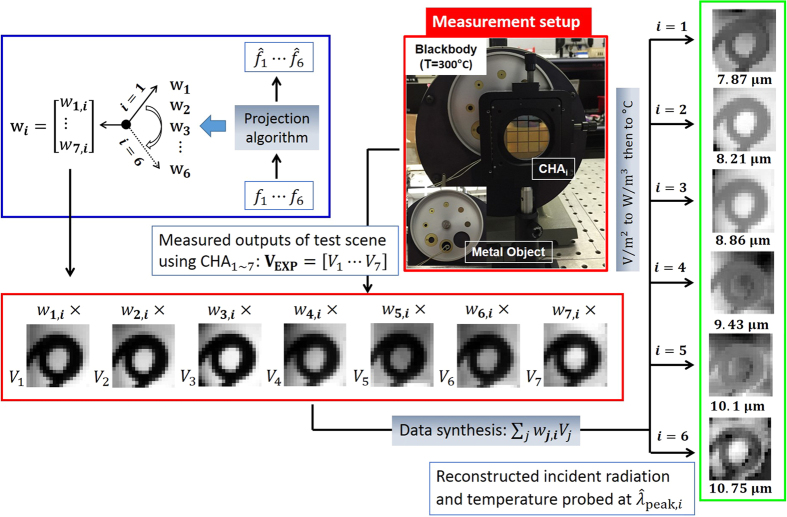
Reconstructed multispectral LWIR images of a metal ring object. The spatial and spectral properties were reconstructed using six synthesized spectral filters (shown in the top left panel of Fig. 3) by means of our detection strategy (using seven SP-filters array) at six different wavelengths (7.87, 8.21, 8.86, 9.43, 10.1, 10.75 μm).

Further evaluation was carried out by creating a different test scenario with a metal object placed between a blackbody and CHA_*i*_. In the measurement, seven output images 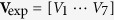 as shown in Fig. 4, were captured as the seven SP filters (CHA_1~7_) were sequentially placed in front of a new test scene. Each output image shows a metal ring object under blackbody illumination. Note that the LWIR camera records a 320 × 240 pixel image, however the region that captured the metal coil consists of 10 × 10 pixels. For reconstructions at
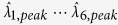
, the same sets of weights 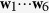
were applied and each was synthesized with**V**_exp_. Resulting from weighted synthesis, the six reconstructed images were obtained including the spatial and spectral properties of a metal ring under the blackbody background. Images at 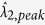 = 8.21 μm and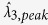 = 8.86 μm indicate good reconstructions, which is attributed to the low projection errors (4.5% and 5.7%) in the preprocessing of our detection strategy. On the other hand, an artifact was observed in the image at 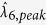 = 10.75 μm, showing a poor reconstruction. This is because the quality of synthetic filter 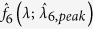 is not of sufficient quality, since the projection error 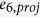 is relatively high, at almost 20% compared to the desired spectral filter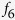. The limitation of synthetic filter approximation was clearly evident beyond 10.75 μm because there was a lack of spectral responsivities or information provided by seven SP filters. To improve the quality, the resonance range of SP-filter structures must be extended to longer wavelengths and we expect this will resolve artifacts, such as the one observed at 10.75 μm.

### Multispectral detection of an extremely hot object using proposed plasmonic-algorithmic spectrometer

**Figure 5:**
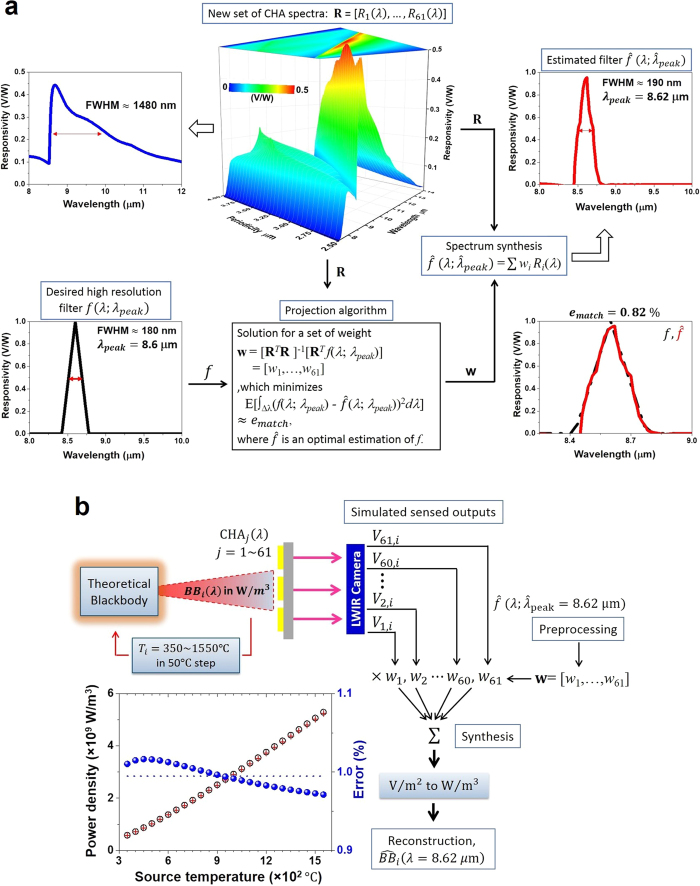
Proposed plasmonic-algorithmic spectrometer to detect objects at extremely high temperature. (**a**) Preprocessing using a set of SP-spectral responsivities 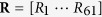 to design a triangular narrowband filter 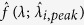 with 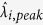 = 8.62 μm and a high resolution FWHM = 190 nm. (**b**) Successful reconstructions (reconstruction error,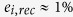) of source radiations at 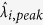 = 8.62 μm as temperature varied from 350 to 1550 °C with a step of 50 °C.

The proposed target detection strategy (based on the fabricated SP filter arrays and algorithmic spectrometer) was successfully demonstrated for a simple thermal source and a metal ring object. This success motivated us to apply the detection strategy towards target detection under extreme conditions (e.g., target temperature ≥1000 °C). To create a test scenario, an IR radiation source was again assumed to be unknown and characterized by Planck’s law with the blackbody temperature varied from 350 to 1550 °C in 50 °C steps, and moreover we assumed that the plasmonic detector system is used for remote sensing of an extremely hot object (i.e., our detector system is not directly exposed to the extremely high temperature or it is not enclosed in the extremely high temperature environment). For detection purpose, the desired LWIR spectral filter 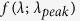
was specified with a triangle shape, a peak at *λ*_*peak*_ = 8.6 μm and a high resolution with FWHM = 180 nm. By preprocessing, this filter was estimated to be 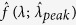 with 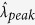 = 8.62 μm and FWHM = 190 nm using a set of 61 SP-spectral responsivities,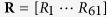 as shown in Fig. 5a (Note: Simulation of 61 SP-spectra is available in the Supporting Information section). The corresponding weight set, **w** was obtained and the projection error was 0.82%. For each target (blackbody) temperature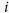, SP-outputs 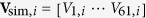 were obtained, and then linearly synthesized with weights **w** for reconstructing the source radiation at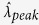 = 8.62 μm. Figure 5b shows that the reconstruction is in good agreement with the true values in extremely high temperature range.

## Discussion

The capability of synthesizing spectral filters using plasmonic spectral filter arrays for LWIR target detection was demonstrated. For the experiment, seven different plasmonic structures based on a circular hole array were fabricated on a GaAs wafer. Band-pass filtered responsivity curves from the plasmonic filter arrays were measured showing a first-order SP resonance tuned in the 7.5–11 μm range. A new concept in IR sensing technique called algorithmic spectrometry (or the IR retina concept) was applied to decorrelate the spectral redundancy present in the sensed data, as the responsivity curves of the plasmonic filter arrays are partially overlapping. The decorrelation process was done by performing a weighted linear synthesis among output images captured by an LWIR camera as seen through the plasmonic filter arrays. The synthetic outputs reconstructed incident radiation from targets at a specified LWIR wavelength location. For demonstration, radiation from a blackbody and a metal object were reconstructed successfully at different wavelength locations 7.87–10.75 μm. Moreover, the potential application of plasmonic filter arrays for detecting targets at extremely high temperature was addressed by reconstructing a radiation of target in the range of 350–1550 °C.

## Methods

### Fabrication procedure of SP filter array based on CHA structure

We fabricated a single gold layer perforated two-dimensional square array of circular hole arrays (CHAs) with seven different periods (*p* = 2.0 mm to 3.2 μm with a step of 0.2 μm; the size of each CHA-units is 1 × 1 cm^2^) on a double-side polished 350 μm-thick, 2-inch semi-insulating GaAs wafers with etch pit density (EPD) of <5000. The bare wafer was cleaned with a standard wet-cleaning procedure using acetone, methanol, and deionized (DI) water for three minutes for each step, followed by a nitrogen blow-off. For good adhesion of AZ5206 photoresist (PR) to the wafer, hexamethyldisilazane (HMDS) was spin-coated using a spinner at 5K rpm for 30 s and then the wafer was hotplate-baked at 100 °C for 60 s. After that, the wafer was spin-coated with AZ5206 PR at 5K rpm for 50 s and baked on a hot plate at 100 °C for 60 s. Conventional photolithography process was then employed to precisely pattern the CHAs on the wafer surface using a Karl Suss MJB-3 mask aligner with a UV mercury lamp (200 W). The wafer was exposed to the UV radiation with an exposure density of 6 mW/cm^2^ for 23 s. The exposed wafer was hotplate-baked at 112 °C for 90 s and flood-exposure was performed for 200 s to reverse the polarity of the positive PR to the negative, which is known as image reversal process (This image reversal process readily enabled fabricating small-featured patterns, facilitating a lift-off process). The image reversal processed sample was developed in a diluted developer (AZ340 developer:DI water = 1:6) for 40 s. For metallization of the CHAs, two metal layers of Ti (5 nm)/Au (100 nm) were deposited on the wafer by an electron-beam evaporator. Ti was used to enhance the adhesion between Au and GaAs in the metallization process. Then, a standard lift-off process using acetone was applied to form the CHAs. Additional Ti metal layer (200 nm) was deposited by an electron-beam evaporator on area that has not been shaped CHAs.

### FTIR transmission measurement

The transmission of the fabricated SP filter array consisting of CHAs with seven different periods was measured using a Thermo Scientific Nicolet 5700 FTIR spectrometer under nitrogen atmosphere of 30~40 sccm to prevent infrared absorption by air. The transmission measurement was carried out at normal incidence to each CHA-units and was normalized to a single beam transmission spectrum of a bare GaAs wafer which was first measured as a background spectrum. As expected, the experimental results showed that the SP resonance wavelength were red-shifted with increasing the periodicity of the CHAs (Fig. 1c).

## Additional Information

**How to cite this article**: Jang, W.-Y. *et al.* Experimental Demonstration of Adaptive Infrared Multispectral Imaging using Plasmonic Filter Array. *Sci. Rep.* **6**, 34876; doi: 10.1038/srep34876 (2016).

## Supplementary Material

Supplementary Information
