# Supplementary material for: Experimental Demonstration of Adaptive Infrared Multispectral Imaging using Plasmonic Filter Array
Source: Sci Rep. 2016 Oct 10;6:34876. doi: 10.1038/srep34876 (PMC5056518; doi:10.1038/srep34876)
Supplement: Supplementary Information [file srep34876-s1.pdf]

**Supporting Information:**

# Experimental Demonstration of Adaptive Infrared Multispectral Imaging using Plasmonic Filter Array

*Woo-Yong Jang[1,2,*,+], Zahyun Ku[3,+], Jiyeon Jeon[4], Jun Oh Kim[4], Sang Jun Lee[4], James Park[1], Michael J. Noyola[1] and Augustine Urbas[3]*

## Projection algorithm for the design of synthetic spectral filter

Suppose that an arbitrary target of interest whose spectral responsivity in the long-wave infrared (LWIR) is denoted by the function $f_i(\lambda;\lambda_i)$ where $\lambda_i$ is the peak wavelength. Also suppose that a broadband LWIR camera with a responsivity spectrum $R_{LWIR}$ is used to measure the illuminated object via several different plasmonic filter array. The transmission spectrum of the $i^{\text{th}}$ plasmonic filter in the array is denoted by $T_k$. Then the transmitted output at the camera can be represented by the product $R_k(\lambda) = T_k(\lambda) \cdot R_{LWIR}(\lambda)$, which is considered as the responsivity via each $T_k$. Let's consider $f_i(\lambda;\lambda_i)$ to be a desired or hypothetical and high resolution narrowband LWIR spectral filter. Imagine that such a filter $f_i$ placed in front of a LWIR broadband camera would be used as an apparatus or a sort of spectrometer for measuring and recording the spectrum of target-of-interest in a scene at wavelength $\lambda_i$. To realize this filter $f_i$, the underlying idea is to approximate $f_i$ by optimally synthesizing a group of detector's responsivity spectrum in $\mathbf{R}$ = $[R_1(\lambda), \dots, R_k(\lambda)]$ using a set of weights in $\mathbf{w}_i$ (i.e., $\hat{f}_i(\lambda;\lambda_i) = \sum_{j=1}^{k} w_{i,j} R_j(\lambda)$ where $\mathbf{w}_i = [w_{i,1}, \dots, w_{i,k}]$). One

avenue to calculate $\mathbf{w}_i$ is to project $f_i$ to the space spanned by $\mathbf{R}$. The projection algorithm to solve for $\mathbf{w}_i$ is provided as follows. Let us define the photocurrent $I_i$ as the detector output which is proportional to the integral $\int_{\lambda_{min}}^{\lambda_{max}} p(\lambda) f_i(\lambda; \lambda_i) d\lambda$ as a target with a given spectrum $p(\lambda)$ is sensed by $f_i$. In radiometry, this source spectrum $p(\lambda)$ is termed the IR spectral irradiance at the detector's active area. By drawing freely from ref. 19, the approximation for $f_i$ is carried out by minimizing the wavelength-integrated minimum mean square error between $I_i$ and $\hat{I}_i$ (i.e., $|I_i - \hat{I}_i|^2$ ) where $\hat{I}_i$ is proportional to $\int_{\lambda_{min}}^{\lambda_{max}} p(\lambda) \hat{f}_i(\lambda; \lambda_i) d\lambda$. First, this error can be defined and mathematically expressed using Cauchy-Schwarz inequality as given below,

$$|I_i - \hat{I}_i|^2 \approx \left| \int_{\lambda_{min}}^{\lambda_{max}} p(\lambda) \left[ f_i(\lambda; \lambda_i) - \hat{f}_i(\lambda; \lambda_i) \right] d\lambda \right|^2$$

$$= \left| \int_{\lambda_{min}}^{\lambda_{max}} p(\lambda) \left[ f_i(\lambda; \lambda_i) - \sum_{j=1}^{k} w_{i,j} R_j(\lambda) \right] d\lambda \right|^2$$

$$\leq \left\{ \int_{\lambda_{min}}^{\lambda_{max}} \left| p(\lambda) \left[ f_i(\lambda; \lambda_i) - \sum_{j=1}^{k} w_{i,j} R_j(\lambda) \right] \right| d\lambda \right\}^2$$

$$\leq \left[ \int_{\lambda_{min}}^{\lambda_{max}} p^2(\lambda) d\lambda \right] \left\{ \int_{\lambda_{min}}^{\lambda_{max}} \left[ f_i(\lambda; \lambda_i) - \sum_{j=1}^{k} w_{i,j} R_j(\lambda) \right]^2 d\lambda \right\} \tag{S1}$$

Since in most sensing problem, the irradiance information is typically unknown and the goal is to reconstruct and identify what it is, the weight calculation should not depend on $p(\lambda)$. Then minimizing the maximum of $|I_i - \hat{I}_i|^2$ is equivalent to the minimization of the second integral which is the error between $f_i$ and $\hat{f}_i$,

$$|I_i - \hat{I}_i|^2 \approx \int_{\lambda_{min}}^{\lambda_{max}} \left[ f_i(\lambda; \lambda_i) - \sum_{j=1}^{k} w_{i,j} R_j(\lambda) \right]^2 d\lambda$$

$$= \left|f_i - \hat{f}_i\right|^2 \quad \text{(S2)}$$

, considering that the first integral for the irradiance $p(\lambda)$ is unity. Note that the integral for $\left|f_i - \hat{f}_i\right|^2$ can be estimated by the discretization as

$$\left|f_i - \hat{f}_i\right|^2 = \int_{\lambda_{min}}^{\lambda_{max}} \left[ f_i(\lambda;\lambda_i) - \sum_{j=1}^{k} w_{i,j} R_j(\lambda) \right]^2 d\lambda$$

$$\approx \frac{\Delta\lambda}{N} \sum_{m=1}^{N} \left[ f_i(\lambda_m;\lambda_i) - \sum_{j=1}^{k} w_{i,j} R_j(\lambda_m) \right]^2 \quad \text{(S3)}$$

where $\Delta\lambda = \lambda_{max} - \lambda_{min}$ and $N$ is the number of bins used for discretization. Simplifying Eqn. S3 in matrix form, it is re-written as follows,

$$\left|f_i - \hat{f}_i\right|^2 \approx \frac{\Delta\lambda}{N} \sum_{m=1}^{N} \left[ f_i(\lambda_m;\lambda_i) - \sum_{j=1}^{k} w_{i,j} R_j(\lambda_m) \right]^2 = \frac{\Delta\lambda}{N} \|\boldsymbol{f_i} - \mathbf{R}\mathbf{w}_i\|^2 \quad \text{(S4)}$$

where $\boldsymbol{f_i}$ is the matrix form of the desired filter $f_i(\lambda;\lambda_i)$. Now then the problem is to minimize the mean square error $\left|f_i - \hat{f}_i\right|^2$ in Eqn. S4 to obtain the optimal weight vector $\mathbf{w}_i$ for approximation,

$$e = \left|f_i - \hat{f}_i\right|^2$$

$$= \frac{\Delta\lambda}{N} \|\boldsymbol{f_i} - \mathbf{R}\mathbf{w}_i\|^2$$

$$= \frac{\Delta\lambda}{N} (\boldsymbol{f_i} - \mathbf{R}\mathbf{w}_i)^T (\boldsymbol{f_i} - \mathbf{R}\mathbf{w}_i)$$

$$= \frac{\Delta\lambda}{N} \left( \boldsymbol{f_i}^{\boldsymbol{T}} \boldsymbol{f_i} - \boldsymbol{f_i}^{\boldsymbol{T}} \mathbf{R}\mathbf{w}_i - \mathbf{w}_i^{\boldsymbol{T}} \mathbf{R}^{\boldsymbol{T}} \boldsymbol{f_i} + \mathbf{w}_i^{\boldsymbol{T}} \mathbf{R}^{\boldsymbol{T}} \mathbf{R}\mathbf{w}_i \right) \quad \text{(S5)}$$

Minimize $e$ with respect to $\mathbf{w}_i^{\boldsymbol{T}}$ and solve for $\mathbf{w}_i$

$$0 = -\mathbf{R}^{\boldsymbol{T}} \boldsymbol{f_i} + \mathbf{R}^{\boldsymbol{T}} \mathbf{R}\mathbf{w}_i$$

$$\mathbf{R}^{\boldsymbol{T}} \mathbf{R}\mathbf{w}_i = \mathbf{R}^{\boldsymbol{T}} \boldsymbol{f_i}$$

$$\mathbf{w}_i = (\mathbf{R}^T\mathbf{R})^{-1}\mathbf{R}^T\boldsymbol{f}_i \tag{S6}$$

The calculated weight vector $\mathbf{w}_i$ enables the optimal approximation of $\boldsymbol{f}_i$ by projecting it onto the function space specified by detector spectra $\mathbf{R} = [R_1(\lambda), \dots, R_k(\lambda)]$.

## Simulation of theoretical outputs $V_{theo}$ and desired spectral reconstruction $P_{f_{i,peak}}$

To clarify the quality of measured outputs $\mathbf{V}_{\mathrm{exp}}$ via SP spectral filter array (i.e., seven spectral filters, $\mathbf{CHA}_1$ to $\mathbf{CHA}_7$), theoretical outputs $\mathbf{V}_{\mathrm{theo}} = [\mathrm{V}_{1,\mathrm{theo}} \cdots \mathrm{V}_{7,\mathrm{theo}}]$ were also generated as a blackbody radiation was transmitted through each SP filter and collected at the LWIR camera. First, the Planck's law was used to specify the spectral exitance from a blackbody source,

$$\mathrm{BB}(\lambda) = [BB(\lambda_1), \dots, BB(\lambda_N)] \tag{S7}$$

where each $BB(\lambda_m) = \frac{2\pi hc^2}{\lambda_m^5}\frac{1}{\left(e^{hc/\lambda_m \kappa_B T} - 1\right)}$ and temperature $T$ was set to 300°C since this value was used for the experiment. Considering the radiometry, each collected output $\mathrm{V}_{i,\mathrm{theo}}$ via $\mathbf{CHA}_i$ is realized by the relationship,

$$\mathrm{V}_{i,\mathrm{theo}} = \int_{\lambda_1}^{\lambda_N} \mathrm{BB}(\lambda) \cdot \mathrm{T}_i(\lambda) \cdot \mathrm{R}_{LWIR}(\lambda)\, d\lambda$$

$$\approx \frac{\Delta\lambda}{N}\sum_{m=1}^{N}[BB(\lambda_m) \times T_i(\lambda_m) \times R_{LWIR}(\lambda_m)] \tag{S8}$$

where $\mathrm{T}_i(\lambda)$ and $R_{LWIR}(\lambda)$ are a transmission spectrum of $\mathbf{CHA}_i$ and a responsivity of LWIR camera, respectively. This discrete sum defines the filtered incidence of a blackbody source

via each **CHA**$_i$ on the LWIR camera. In addition, the reconstructed radiation $P_{f_{i,peak}}$ resulting from sampling the true blackbody radiation $\mathrm{BB}(\lambda)$ by ideal spectral filter $f_i$ was obtained by

$$P_{f_{i,peak}} = \frac{\int_{\lambda_1}^{\lambda_N} \mathrm{BB}(\lambda) \cdot f_i(\lambda; \lambda_i)\, d\lambda}{\mathrm{A}_{f_i}}$$

$$\approx \frac{\frac{\Delta\lambda}{N} \sum_{m=1}^{N} [BB(\lambda_m) \times f_i(\lambda_m; \lambda_i)]}{\mathrm{A}_{f_i}} \tag{S9}$$

where $\mathrm{A}_{f_i} = \int f_i(\lambda; \lambda_i)\, d\lambda$ (i.e., the area under the spectral responsivity curve of filter $f_i$) and is a relating factor to yield the power per unit area per wavelength ($W/m^3$). The value $P_{f_{i,peak}}$ is used as a ground truth for accurate comparison in order to measure the quality of experimental reconstruction $\hat{P}_{\hat{f}_{i,peak}}$ using synthetic spectral filter $\hat{f}_i$.

## FTIR-measured transmission spectra and LWIR spectral responsivities via SP filter arrays

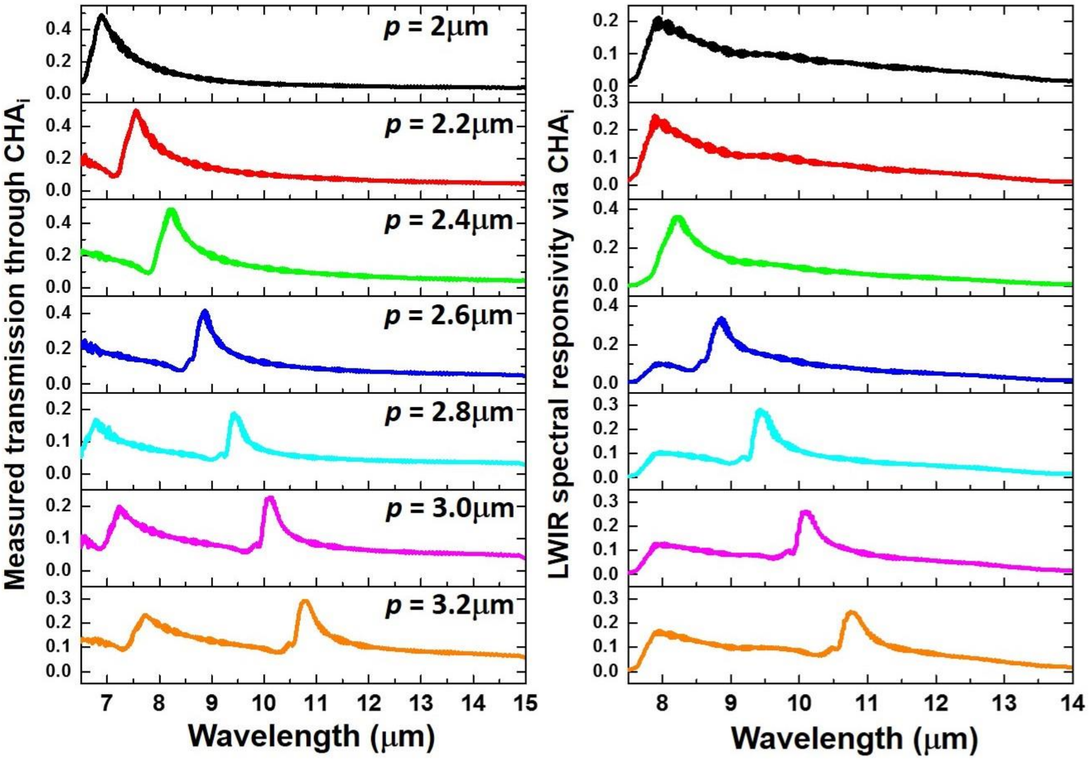


**Figure S1.** (Left) The measured transmission (T*i*) of the fabricated SP filter array (CHA*i*, where the subscript *i* represents the CHA's periodicity, i.e., *p* = 2 μm to 3.2 μm with a step of 0.2 μm) using a Thermo Scientific Nicholet 5700 FTIR spectrometer. (Right) Corresponding LWIR spectral responsivity (R*i*), camera output due to IR beam transmitted via CHA*i* can be calculated with $R_i = T_i \cdot R_{LWIR}$, where $R_{LWIR}$is the relative responsivity of LWIR camera used here.

## Simulation of CHA transmission spectra for the design of high resolution synthetic spectral filter

To design a desired bandpass spectral filter $f(\lambda; \lambda_{peak})$ with a peak wavelength $\lambda_{peak}$ of 8.6

μm and a fine spectral resolution (i.e., FWHM ≤ 200 nm), a new set of 61 CHA's transmission spectra **T** = $[T_1(\lambda), \ldots, T_{61}(\lambda)]$ was simulated. These simulated transmission spectra are to be used to characterize the spectral responsivities **R** = $[R_1(\lambda), \ldots, R_{61}(\lambda)]$ of sensed outputs based on the following relationship, $R_i(\lambda) = T_i(\lambda) \cdot R_{LWIR}(\lambda)$ where $R_{LWIR}(\lambda)$ is the broadband spectral responsivity of LWIR camera. Basically, each $R_i(\lambda)$ represents the amount of source radiation in the LWIR collected on the camera after transmitting from each CHA filter with $T_i(\lambda)$. Using **R** as a set of basis-functions in the projection algorithm, the desired $f(\lambda; \lambda_{peak})$ is approximated by forming a linear combination of $R_i$ in **R**. As a result, the algorithm generated the synthetic spectral filter $\hat{f}(\lambda; \hat{\lambda}_{peak})$ with $\hat{\lambda}_{peak}$ = 8.62 μm and FWHM = 190 nm. Description of how to model transmittance spectra ***T*** is provided as follows. First, the CHA structure as shown in the left panel of Figure S2 was specified by having an array of Au-based circular holes developed on a GaAs substrate. Next optimal geometrical parameters/material properties for the CHA structure need to be determined. For the geometry of CHA, the ratio ($r$ = $d/p$) between the diameter of the hole aperture $d$ and the pitch $p$ was varied. In Figure S2 (Right panel), several transmission curves were obtained as the ratio $r$ was increased from 0.5 to 0.8 by varying $d$ while a pitch $p$ was fixed at 2 μm. Measured transmission curve shown in Figure S2 (Right panel) was also plotted by blue dotted line as a reference and was used to validate the resonant wavelengths (or peak locations) and shapes of the simulated transmission curves. By comparison, the transmission spectrum with $r$ = 0.6 fits the measurement.

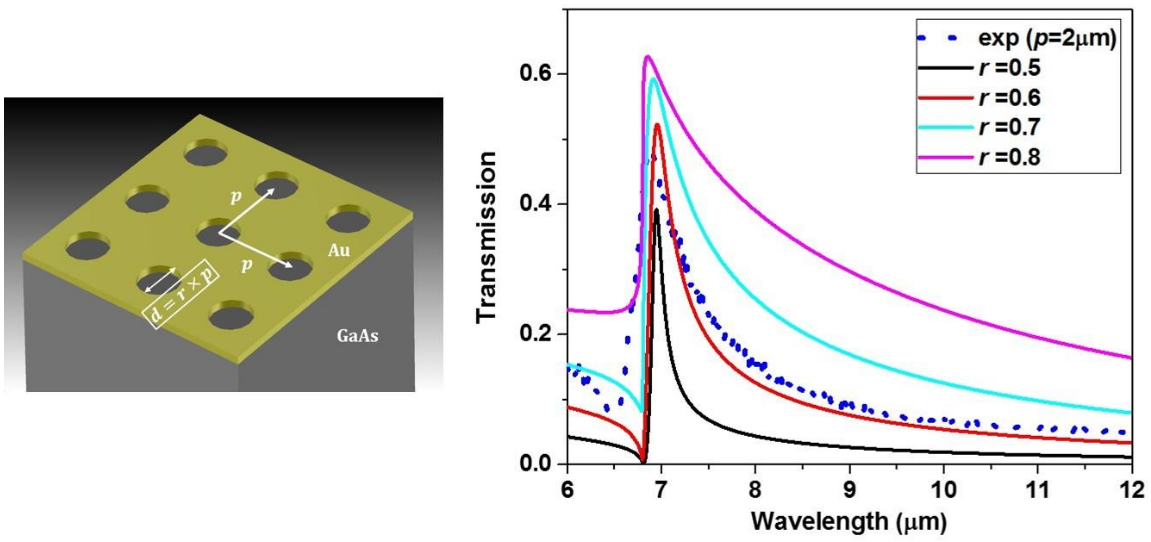


**Figure S2.** (Left) Illustration of CHA structure. (Right) Transmission depending on the ratio $d/p$ ($d$ is the diameter of aperture) varying from 0.5 to 0.8, where a pitch $p$ is fixed at 2 μm.

Then, for the material property of Au, Drude model was applied by using the fitting parameter $\gamma$ for the permittivity of Au (Left panel of Figure S3). As $\gamma$ was varied from 1.4 to 3.4, the corresponding transmission spectra in Figure S3 (Right panel) were obtained and simulated transmission with $\gamma$= 3.4 shows the closest match to the measured spectrum.

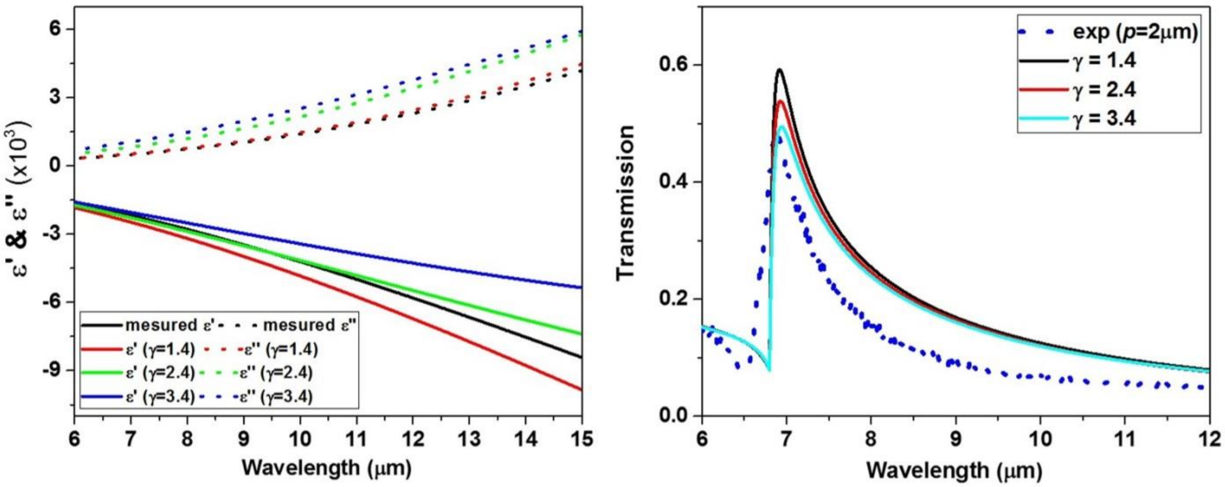


**Figure S3.** (Left) Measured permittivity of Au and fitting parameter ($\gamma$) used in Drude model changing from 1.4 to 3.4. (Right) Simulated transmission of CHA depending on $\gamma$ displayed in left panel.

The material property of a GaAs substrate was also considered and optimized by searching for the transmission spectra of CHA shown in Figure S4 as the refractive index of GaAs was changed from 3.3 to 3.4. Using optimized setting, the transmission spectrum of CHA for $p$ = 2 μm was simulated. Finally, the entire modeling process was repeated by varying pitch $p$ from 2.5 μm to 4 μm in 25 nm step. As a result, a group of 61 different transmission spectra $\boldsymbol{T}$ was obtained as displayed in Figure S5.

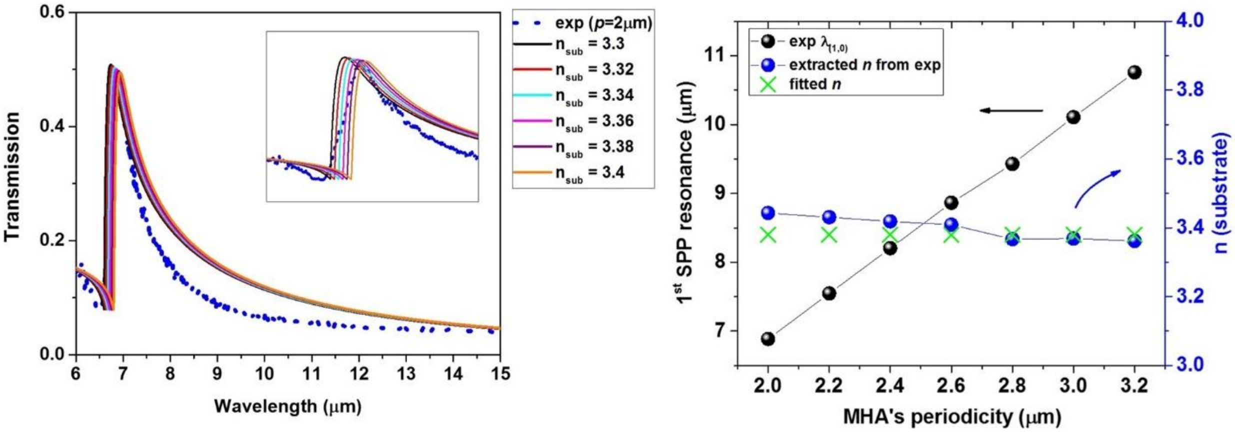


**Figure S4.** (Left) Transmission of CHA with varying the refractive index of GaAs substrate from 3.3 to 3.4 with a step of 0.02. (Right) Real part of refractive index of GaAs substrate which can be extracted from experiment using SP dispersion relation (i.e. the ratio of 1st SPP resonance wavelength to periodicity in CHA, $n = \lambda_{(1,0)}/p$) as compared with Re(n) found using simulation as shown in left panel.

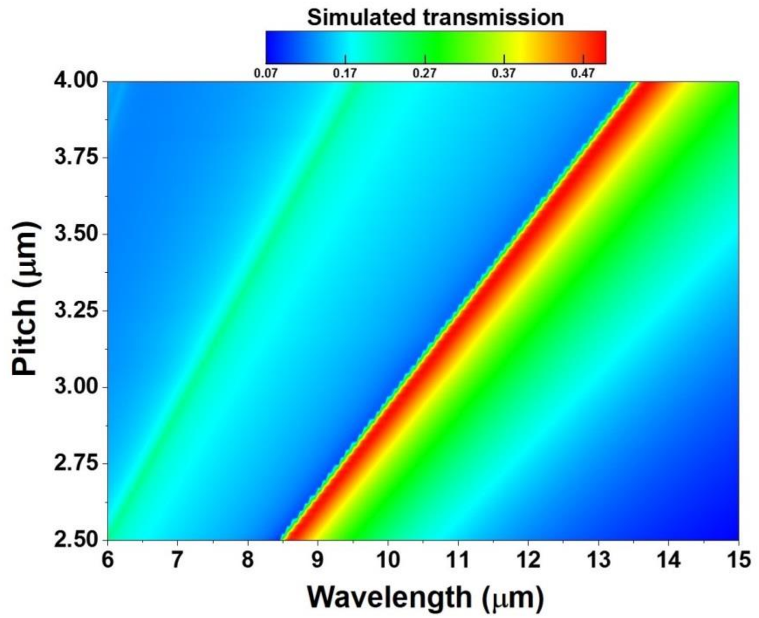


**Figure S5.** The colormap shows the simulated transmission spectra of CHA structure as a function of pitch *p*. The pitch *p* was varied from 2.5 μm to 4 μm with a step of 25 nm. The color changes from blue (0.07) to red (0.502) showing the transmission level. As expected, two lowest resonances (1st and 2nd SPP resonances) can be clearly seen in the colormap.
